# A self-powered UV photodetector based on TiO_2_ nanorod arrays

**DOI:** 10.1186/1556-276X-8-188

**Published:** 2013-04-23

**Authors:** Yanru Xie, Lin Wei, Guodong Wei, Qinghao Li, Dong Wang, Yanxue Chen, Shishen Yan, Guolei Liu, Liangmo Mei, Jun Jiao

**Affiliations:** 1 School of Physics and State Key Laboratory of Crystal Materials, Shandong University, Jinan 250100, People's Republic of China; 2 School of Information Science and Engineering, Shandong University, Jinan, 250100, People's Republic of China; 3 Department of Mechanical and Materials Engineering, Portland State University, P.O. Box 751, Portland, OR, 97207-0751, USA; 4 Department of Physics, Portland State University, P.O. Box 751, Portland, OR, 97207-0751, USA

**Keywords:** TiO_2_, Nanorod, Ultraviolet photodetector, Solid–liquid heterojunction

## Abstract

Large-area vertical rutile TiO_2_ nanorod arrays (TNAs) were grown on F/SnO_2_ conductive glass using a hydrothermal method at low temperature. A self-powered ultraviolet (UV) photodetector based on TiO_2_ nanorod/water solid–liquid heterojunction is designed and fabricated. These nanorods offer an enlarged TiO_2_/water contact area and a direct pathway for electron transport simultaneously. By connecting this UV photodetector to an ammeter, the intensity of UV light can be quantified using the output short-circuit photocurrent without a power source. A photosensitivity of 0.025 A/W and a quick response time were observed. At the same time, a high photosensitivity in a wide range of wavelength was also demonstrated. This TNA/water UV detector can be a particularly suitable candidate for practical applications for its high photosensitivity, fast response, excellent spectral selectivity, uncomplicated low-cost fabrication process, and environment-friendly feature.

## Background

Ultraviolet (UV) photodetector has been a popular research issue for its potential applications in a wide range of fields, such as remote control, chemical analysis, water purification, flame detection, early missile plume detection, and secure space-to-space communications [1]. To avoid the use of filters and achieve visible-blind operation, wide bandgap semiconductors, such as GaN, SiC, ZnO, and TiO_2_[2-8], have been studied during the last decade for wide-spreading usage in photodetection, especially in the ultraviolet region. Among conventional available UV photodetectors, quite many kinds of structures have been fabricated, which in most cases are based on epitaxial growth process and various solid-state junction structures. Typical examples are photodetectors based on p-n junction, p-i-n photodiodes, Schottky barrier (SB), metal–semiconductor-metal, and metal-insulator-semiconductor structures [9-15]. These photodetectors typically require an external bias as the driving force to prevent the recombination of photogenerated electron–hole pairs. For large-area two-dimensional arrays that contain huge amounts of small UV sensors, energy supply will be one of the main challenges for such sensor systems.

Recently, self-powered nanodevices and nanosystems have attracted lots of attention due to their various advantages. Xu et al. fabricated a nanowire pH sensor and a nanowire UV sensor powered by a piezoelectric nanogenerator equipped with a capacitor, demonstrating a self-powered system composed entirely of nanowires [16]. Yang et al. reported a self-powered ultraviolet photodetector based on a single Sb-doped ZnO nanobelt bridging an ohmic contact and a Schottky contact, in which high photoresponse sensitivity and short response time were observed [17]. Bai et al. reported a ZnO nanowire array ultraviolet photodetector with self-powered properties, in which a high sensitivity of 475 without external bias is found [18]. Although n-type semiconducting ZnO is a significant material for optoelectronic applications, it is unstable under both acidic and alkaline conditions. Also, the photoresponse of ZnO-based UV detector is sensitive to the surrounding atmosphere and can be easily affected by oxygen as well as water molecules. On the other hand, TiO_2_ nanostructures have also emerged as very promising materials for optoelectronic devices due to their excellent physical and chemical properties, such as high melting point, chemical inertness, physical stability, direct bandgap (rutile 3.0 eV), high photoconversion efficiency, and photostability. Self-powered UV photodetectors based on a photochemical cell have been fabricated using a liquid I^-^/I_3_^-^ redox couple electrolyte and a nanocrystalline TiO_2_ film [19] or a multilayer TiO_2_ nanorod-assembled cloth/nanorod array-based electrode [20]. Impressive performances were observed in these UV detectors. However, liquid I^-^/I_3_^-^ redox couple electrolyte is not ideal for long-term operation: it is highly corrosive, volatile, and photoreactive, interacting with common metallic components and sealing materials. From this point, water-based electrolytes may be the safest, most stable, and most environment-friendly electrolyte. Lee et al. reported a UV detector based on TiO_2_/water solid–liquid heterojunction [21]. This self-powered UV photodetector behaves similar to a Schottky diode and works in photovoltaic mode. Moreover, TiO_2_/water solid–liquid heterojunction UV detector exhibits high photosensitivity, excellent spectral selectivity, linear variations in photocurrent, and fast response. Cao et al. reported the photocurrent response of TiO_2_ nanorod arrays under UV illumination using a 0.5 M Na_2_SO_4_ aqueous electrolyte [22], in which TiO_2_ nanostructures can harvest more incident light photons compared to a flat thin-film active layer because of the markedly enlarged TiO_2_/electrolyte contact area. However, they did not report its photosensitivity and spectral response. All of these reported results indicate that self-powered UV detectors based on TiO_2_ nanostructures show great potential as excellent candidates for commercial UV photodetectors. Further advancements for TiO_2_-based self-powered UV detectors demand a deeper understanding of the main parameters determining the photoelectric behavior, which also requires additional research and insight into the electrical transporting process in these nanostructured devices.

In this paper, self-powered UV detectors were fabricated based on single-crystalline rutile TiO_2_ nanorod arrays (TNAs), which were grown directly on fluorine-doped tin oxide (FTO) glass by a low-temperature hydrothermal method. This UV photodetector establishes a built-in potential due to its Schottky barrier-like behavior. The built-in potential separates the electron–hole pairs generated by UV light and makes the photodetector generate photocurrent without any external bias. A considerable photocurrent response was observed under UV light illumination. Also, this self-powered photodetector demonstrates fast photoresponse speed, high photosensitivity, excellent spectral selectivity, uncomplicated low-cost fabrication process, and environment-friendly feature.

## Methods

### Growth of TiO_2_ nanorod arrays by hydrothermal process

The single-crystalline rutile TNAs used for this study were grown vertically on FTO glass using the following hydrothermal methods: a diluted hydrochloric solution was prepared by mixing 50 mL of deionized water with 40 mL of concentrated hydrochloric acid and was stirred at ambient temperature for 5 min, and then 400 μL of titanium tetrachloride was added to the mixture. After being stirred for another 10 min, the mixture was injected into a stainless steel autoclave with a Teflon container cartridge. The FTO substrates were ultrasonically cleaned and were placed at an angle against the Teflon container wall with the conducting side facing down. Hydrothermal synthesis was conducted at 180°C for 2 h. After synthesis, the autoclave was cooled to room temperature under flowing water, and the FTO substrates were taken out, rinsed thoroughly with deionized water, and annealed at 500°C for 1 h to improve the crystalline structure.

### Assemble of TNA/water solid–liquid heterojunction

**Figure 1:**
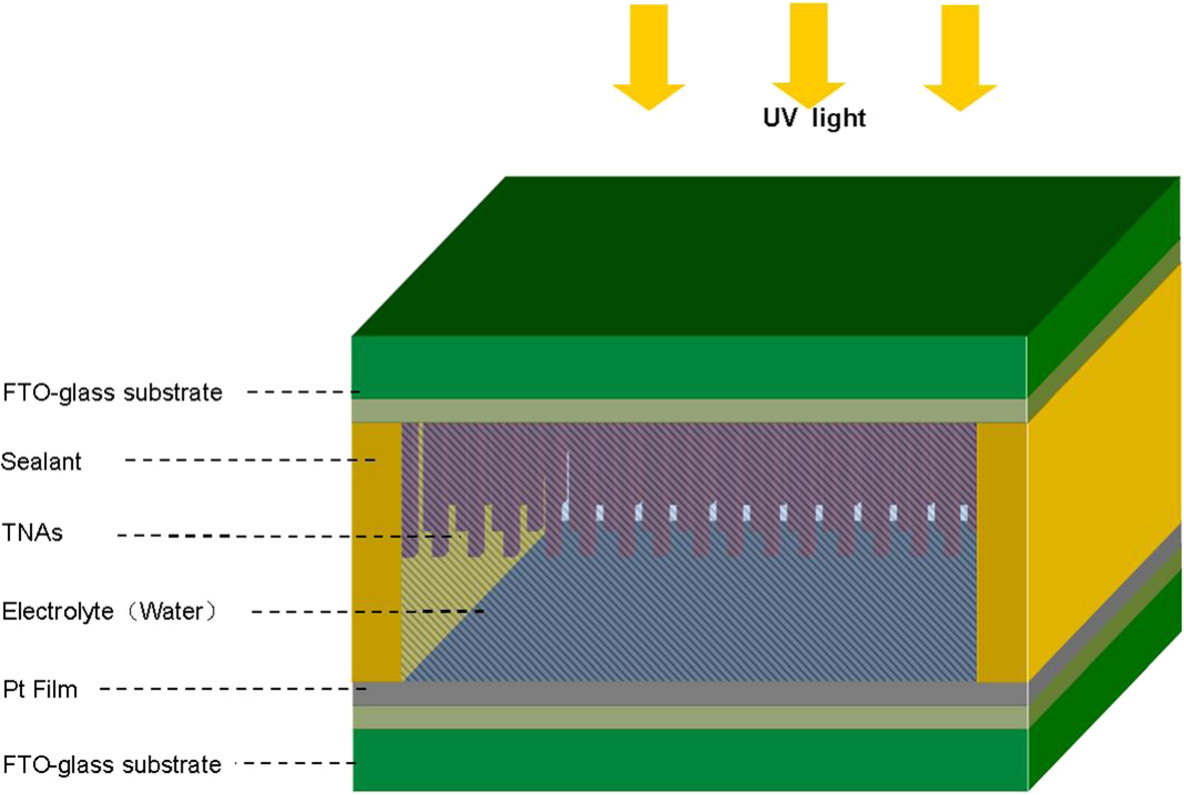
Schematic device structure of the TNA/water heterojunction ultraviolet photodetector.

The schematic structure of the TNA/water solid–liquid heterojunction UV photodetector is shown in Figure 1. For device fabrication, the TNA layer grown on FTO glass was used as the active photoanode. Pt counter electrodes were prepared by depositing a 20-nm Pt film on FTO glass using magnetron sputtering. A 60-μm-thick sealing material (SX-1170-60, Solaronix SA, Aubonne, Switzerland) was pasted onto the Pt counter electrodes. Afterward, the Pt counter electrode and a nanostructure TNA photoanode were sandwiched and sealed with the conductive sides facing inward. Finally, some high-quality deionized water was injected into the space between TNA/FTO glass and Pt/FTO glass electrodes as an electrolyte. A solid–liquid heterojunction UV photodetector was then fabricated, and the active area of the TNA/water device for UV light detection was about 0.126 cm^2^.

### Characterization of the TNA samples and the UV photodetector

The crystal structure of the TNA samples were examined by X-ray diffraction (XRD; XD-3, PG Instruments Ltd., Beijing, China) with Cu Kα radiation (*λ* = 0.154 nm) at a scan rate of 2°/min. The surface morphology was characterized using a scanning electron microscope (SEM; Hitachi S-4800, Hitachi, Ltd., Chiyoda, Tokyo, Japan). The optical transmittance was measured using a UV-visible dual-beam spectrophotometer (TU-1900, PG Instruments, Ltd.). The photoresponse characteristics of the self-powered UV detector in the dark and under illumination were recorded with a programmable voltage–current source (2400, Keithley Instruments Inc., Cleveland, OH, USA). A 500-W xenon lamp (7ILX500, 7Star Optical Instruments Co., Beijing, China) equipped with a monochromator (7ISW30, 7Star Optical Instruments Co.) was used as light source for spectral response characterization. For the photoresponse switching behavior measurement, a UV LED (NCSU033B(T), Nichia Co., Japan) with a wavelength of 365 nm was used as light source, and the photocurrent was obtained by an electrochemical workstation (RST5200, Zhengzhou Shirusi Instrument Technology Co. Ltd, Zhengzhou, China).

## Results and discussion

**Figure 2:**
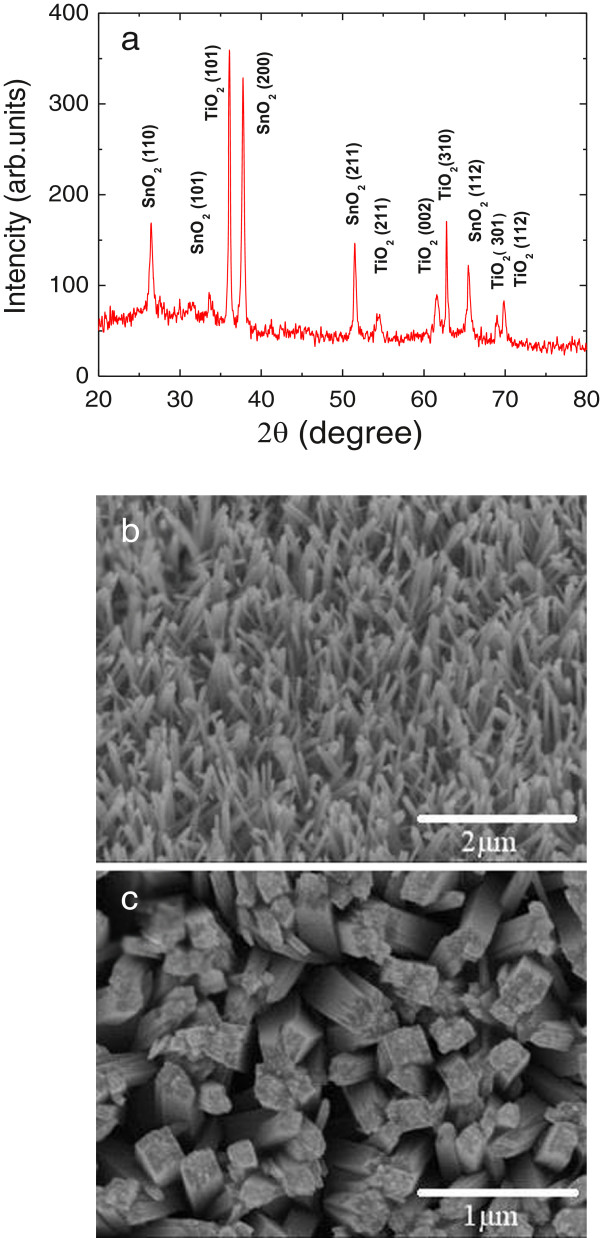
**XRD pattern and SEM images of TiO**_**2 **_**nanorod arrays. **(**a**) X-ray diffraction pattern of the TiO_2_ nanorod array grown on FTO glass. (**b**) SEM image (40° tilted) of the TiO_2_ nanorod array grown on FTO glass by hydrothermal method. (**c**) A high-magnification top-view SEM image of TiO_2 _nanorod array.

The well-aligned TNAs with pure rutile phase are verified by the XRD pattern in Figure 2a. The *θ*-2*θ* scan pattern shows that the TiO_2_ nanorods grown on FTO-coated glass substrates have a tetragonal rutile structure (JCPDS 02–0494). The SnO_2_ peaks are due to the pattern of FTO glass substrate. The reason that the hydrothermal growth method delivers rutile phase instead of other phases, such as anatase and brookite, could be attributed to the small lattice mismatch between FTO and rutile. Both rutile and SnO_2_ have near-identical lattice parameters with *a* = 4.594, *c* = 2.958 Å and *a* = 4.737, *c* = 3.185 Å for TiO_2_ and SnO_2_, respectively, making the epitaxial growth of rutile TiO_2_ on FTO film possible. On the other hand, anatase and brookite have lattice parameters of *a* = 3.784, *c* = 9.514 Å and *a* = 5.455, *c* = 5.142 Å, respectively. The production of these phases is unfavorable due to a very high activation energy barrier which cannot be overcome at the low temperatures used in this hydrothermal reaction. Figure 2b,c shows the micrographs of an as-grown TiO_2_ nanorod array taken by a field emission scanning electron microscope at tilted and top views. The images at different magnifications and at different locations reveal that the entire surface of the FTO-coated glass substrate is uniformly covered with ordered TiO_2_ nanorods. Further analysis indicates that the nanorods are typically 100 to 150 nm in diameter and are tetragonal in shape with square top facets consisting of many small grids. The density of nanorods is typically 20 nanorods/μm^2^. No significant changes in nanorod array morphology were observed after annealing at 500°C.

**Figure 3:**
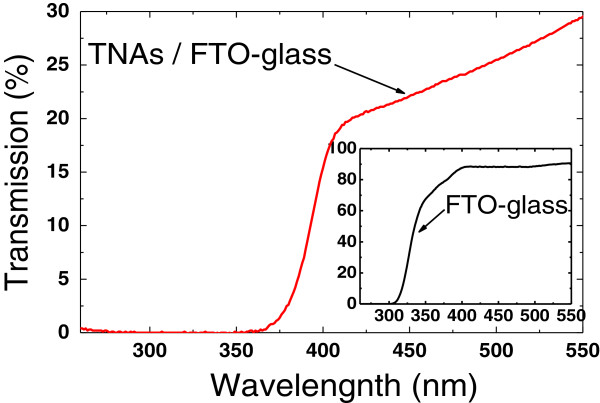
**The UV-visible absorption spectra of TiO**_**2 **_**nanorod array and an FTO glass substrate (inset).**

The optical property of the TNA was investigated using UV-visible transmittance spectrum. Figure 3 shows the optical transmittance spectra of the TNA sample and the FTO glass substrate. An obvious sharp absorption edge can be observed at 420 nm, which can be attributed to the energy bandgap of rutile TiO_2_ nanorods. As the size of the TiO_2_ nanorod is well above the TiO_2_ Bohr exciton diameter, no obvious blueshift caused by quantum confinement is observed. The low transmittance (20% to 30%) in the wavelength ranges of 400 to 550 nm is caused by the strong light scattering from TNAs. An absorption edge for the FTO glass substrate is about 310 nm, as shown in the inset of Figure 3. From these two transmittance spectra, we can conclude that only light with the wavelength between 310 and 420 nm can reach the TNAs and contribute to the UV photoresponsivity, which is confirmed in the following spectral response characterization.

**Figure 4:**
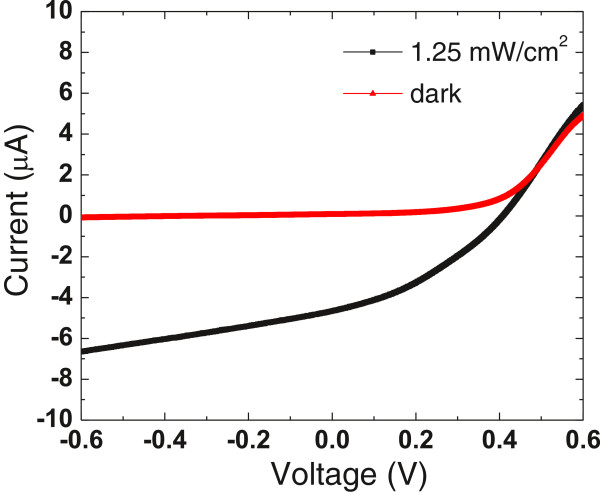
**Current–voltage (*I*-*V*) characteristics of the UV detector. **Typical *I*-*V *curves for the self-powered TNA/water UV detector measured at applied bias from -0.6 to 0.6 V under dark (red line) and 365-nm UV light illumination (black line).

**Figure 5:**
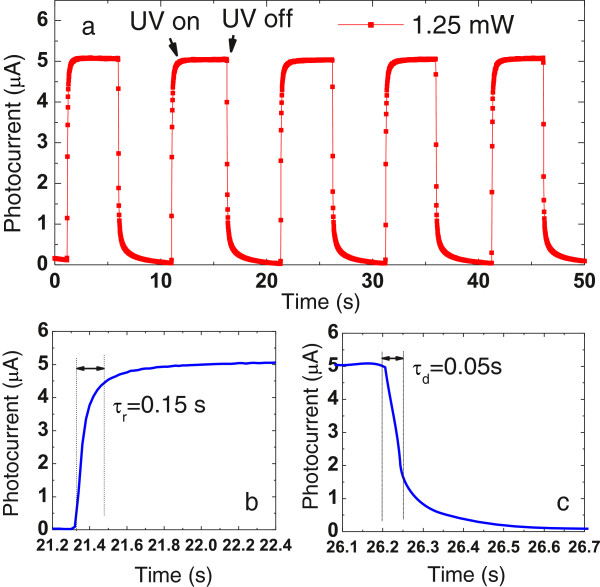
**Time response of the TNA/water UV detector. **(**a**) Photocurrent response under on/off radiation of 1.25 mW/cm^2 ^of UV light illumination. (**b**) Enlarged rising and (**c**) decaying edges of the photocurrent response.

Typical current–voltage (*I*-*V*) characteristics of the UV detector are shown in Figure 4. An SB-like behavior of the UV detector is demonstrated from the dark *I*-*V* curve, which shows a forward turn-on voltage of about 0.4 V and a rectification ratio of about 44 at ± 0.6 V. Under the illumination of 1.25 mW/cm^2^ of UV light (*λ* = 365 nm), the UV detector shows an excellent photovoltaic performance, yielding a short-circuit current of 4.67 μA and an open-circuit voltage of 0.408 V. This inherent built-in potential arises from the SB-like TiO_2_-water interface, acts as a driving force to separate the photogenerated electron–hole pairs, and produces the photocurrent. Therefore, this device can operate not only at photodiode mode but also at photovoltaic mode without any external bias. The real-time photocurrent response of the self-powered UV detector was measured at 0-V bias under a 365-nm UV LED on/off switching irritation with an on/off internal of 5 s. Five repeat cycles under an on/off light intensity of 1.25 mW/cm^2^ are displayed in Figure 5a, in which the photocurrent was observed to be consistent and repeatable. A fast photoresponse can be clearly seen. From enlarged rising and decaying edges of the photocurrent response shown in Figure 5b,c, the rise time and the decay time of the UV detector are approximately 0.15 and 0.05 s, indicating a rapid photoresponse characteristic. On the contrary, TiO_2_ one-dimensional UV photodetectors based on photoconductivity exhibit a much longer recovery time due to the presence of a carrier depletion layer at the nanomaterial surface caused by surface trap states [23]. The photosensitivity of the TNA self-powered UV detector to 365 nm light was also tested using a range of intensities from 12.5 μW/cm^2^ to 1.25 mW/cm^2^. A steadily increasing photocurrent response was observed in relation to increasing incident light intensity (not included here). This UV detector exhibits an excellent capacity to detect very weak optical signals. Even under a weak incident light intensity of 12.5 μW/cm^2^, the magnitude of photosensitivity has already approached two orders.

**Figure 6:**
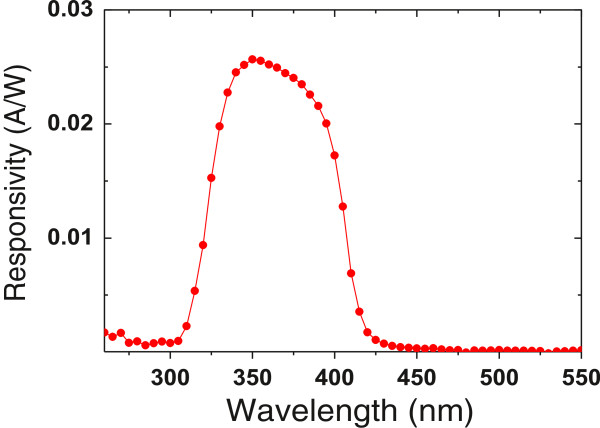
Spectral responsivity characteristic of TNA/water UV photodetector from 260 to 550 nm under 0-V bias.

The wavelength selective ability of the TNA/water UV detector was measured in the range of 260 to 550 nm at 0-V bias, and the result is shown in Figure 6. It is clearly seen that excellent UV light detection selectivity in a spectral range between 310 and 420 nm is observed, which indicates that the device can be used as photodetector for UV-A range (320 ~ 400 nm) application. The maximum responsivity of the spectrum is about 0.025 A/W, located at the wavelength of 350 nm. The spectral response edge of 310 nm is limited by the transmittance of the FTO glass substrate. The edge of 420 nm is attributed to the absorption edge of the TNA layer.

The working principle of the device is discussed simply in the following. When UV light (310 ~ 420 nm) shines on the TNA/water UV detector, the incident photons that pass through the FTO glass into the TNAs and electrons in TiO_2_ are excited from the valence band to the conduction band and then generate electron–hole pairs in the TNAs. The built-in potential produced by solid–liquid heterojunction separates the UV light-generated electron–hole pairs. The separated holes move from the valence band of the TNAs into the interface of TNA/water, subsequently seizing the electrons from the water OH^-^ anions (*h*^+^ + OH^-^ → HO·). Considering the quite large TNA/water surface area, the small diameter of the nanorods, and the built-in interface potential, a fast removal of holes from the surface can be expected. On other hand, the separated electrons transport into the TNA conduction band and are collected easily by the FTO contact as the work function of FTO matches the conduction band of TiO_2_. These electrons move into the external circuit and then come back to the Pt layer of the detector, thereupon returning the electrons to HO· radicals (*e*^-^ + HO· → OH^-^) at the interface of water/Pt. In this way, the built-in potential makes the UV detector generate photocurrent without any external bias. Even though zero bias is applied, the UV detector exhibits high photosensitivity [21,24].

## Conclusions

In conclusion, a photoelectrochemical cell-structured self-powered UV photodetector was developed using water as the electrolyte and a rutile TiO_2_ nanorod array as the active photoelectrode. This device exhibits a prominent performance for UV light detection. Under ambient environment, the photocurrent responses rapidly with UV light on/off switching irradiation. Also, this self-powered TNA/water UV detector demonstrates high photosensitivity and excellent spectral selectivity. All of these results indicate that this novel UV detector can be a promising candidate as a low-cost UV photodetector for commercially integrated photoelectronic applications.

## Competing interests

The authors declare that they have no competing interests.

## Authors’ contributions

The work presented here was performed through the collaboration of all authors. YX carried out the measurements of the TNA/water UV detector and drafted the manuscript. LW conducted the transmittance spectra measurements. GW grew the TNA photoanode. QL carried out the XRD and the SEM characterizations. DW deposited the Pt film and helped fabricate the device. YC supervised the work and finalized the manuscript. SY and GL analyzed the results and participated in the revision of the manuscript. LM and JJ proofread the manuscript and corrected the English. All authors read and approved the final manuscript.
